# Influence of Storage Temperature and Packaging on Bacteria and Yeast Viability in a Plant-Based Fermented Food

**DOI:** 10.3390/foods9030302

**Published:** 2020-03-07

**Authors:** Miriam Cabello-Olmo, María Oneca, Paloma Torre, Jesús Vicente Díaz, Ignacio J. Encio, Miguel Barajas, Miriam Araña

**Affiliations:** 1 Biochemistry Area, Department of Health Science, Public University of Navarre, 31008 Pamplona, Spain; 2 Nutrition and Bromatology Area, Department of Natural Sciences, Public University of Navarre, 31006 Pamplona, Spain; 3 Pentabiol S.L., Polígono Noain-Esquiroz s/n, 31191 Pamplona, Spain

**Keywords:** fermented foods, lactic acid bacteria, packaging, probiotic, storage, temperature, viability, yeasts

## Abstract

Optimization of food storage has become a central issue for food science and biotechnology, especially in the field of functional foods. The aim of this work was to investigate the influence of different storage strategies in a fermented food product (FFP) and further determine whether the regular storage (room temperature (RT) and standard packaging (SP)) could be refined. Eight experimental conditions (four different temperatures × two packaging) were simulated and changes in FFP’s microbial ecology (total bacteria, lactic acid bacteria (LAB), and yeasts) and physicochemical characteristics (pH and moisture content (MC)) were determined following 1, 3, 6, and 12 months. All conditions tested showed a decline in microbial content due to the effect of the temperature, 37 °C being the most detrimental condition, while −20 and 4 °C seemed to be better than RT in some parameters. Vacuum packaging (VP) only had a major effect on MC and we found that VP preserved greater MC values than SP at 3, 6, and 12 months. The correlation analysis revealed that total bacteria, LAB, and yeasts were positively associated, and also both pH and MC showed a correlation. According to our results and with the purpose to maintain the load of viable microorganisms, we observed that the best storage conditions should contemplate SP and freezing or cooling temperature during a period no longer than 3 months.

## 1. Introduction

The development of new functional foods has gained recent interest due to the growing incidence of chronic diseases [1,2] and the central role of nutrition in most of them [3,4]. Among functional foods, fermented foods are recognized as beneficial for humans’ microbiota and are well established in the health market as promising therapeutic agents [5,6,7]. Fermented foods can be defined as foods and beverages produced through the culture of certain microorganisms in controlled conditions [8]. These fermentation processes involve substantial modifications in the food matrix that increase its nutritional value [9,10] and also provide unique organoleptic attributes [11] and useful technological properties [12]. When fermented foods are not subjected to further technological transformations, such as pasteurization or high pressure treatments [13,14], they can be used as vehicle for probiotics: “live microorganisms which when administered in adequate amounts confer a health benefit on the host” [15]. Although recent findings suggest that bacteria viability is not always necessary for producing beneficial clinical effects [16,17,18], major efforts have been directed towards maintaining the highest load of alive microorganisms at the time of consumption.

Although fermentation processes tend to increase food stability [19,20,21], during shelf life food resident microflora must cope with a list of circumstances which endanger their survival. Intrinsic and extrinsic factors that influence on the survival of probiotic species in foods include ingredients, physicochemical characteristics, processing, handling, and storage [22,23,24,25,26,27,28,29]. For instance, acidity is one of the most relevant factors. Most microorganisms grow well at pH around neutral (pH of 7) but an extremely acidic environment is a growth-limiting factor [30,31,32] and is responsible to a large degree for the loss of viability of probiotics [33]. Similarly, nutritional characteristics like water content [34,35], solutes [36], nitrogen [37], or fermentable sugars [29] are relevant aspects to be considered for the microbial metabolism.

Additionally, storage time and temperature can affect the bacterial survival [30,38,39,40] thus the manipulation of environmental temperature could be useful for reducing the loss of viable bacteria. In general, high temperature importantly decreases microorganism’s viability [41,42] while low temperature, like refrigeration, has been reported to be better for the survival of certain probiotics [43]. Other strategies for increasing the survival of microorganisms in foods, focus on minimizing oxygen exposure by manipulating the packaging, incorporating antioxidant compounds, or regulating the environmental light [24,30,44].

Most of the available information relative to probiotic survival comes from studies carried out on dried probiotics [22,42] or dairy products [45,46,47]. Nonetheless, how probiotic bacteria behave in other food matrixes have not been researched in depth. Moreover, because of the increasing demand of lactose-free and vegetarian foods [48], new-era food products have been developed during the last years and alternative food carriers from plant origin are being explored as vehicle for microorganism delivery [41,49]. 

The present research was carried out in a plant-based food, fermented by a combination of lactic acid bacteria (LAB) and yeasts. This food product, henceforth called FFP (fermented food product), is commercialized for animal production as a food supplement with functional properties (HEALTHSTOCK Ref.733627; https://cordis.europa.eu/project/rcn/206082/factsheet/es). Findings from previous studies support that FFP is useful in enhancing performance and immunity in dairy animals [50,51], and a recently published study in a type 2 diabetic rat model revealed its potential anti-diabetic properties [52]. 

In the present manuscript we aimed to determine how storage impacts on the microbial load in FFP and whether different storage conditions alternative to the ongoing one (room temperature and standard packaging) would contribute with a better preservation of the alive microorganisms present in FFP. For this purpose, we conducted a comparative study to determine the influence of different storage conditions (four different temperatures and two packaging conditions) on FFP. Consequently, the findings would allow us to understand the influence of temperature and packaging during FFP storage. 

## 2. Materials and Methods

### 2.1. Raw Material and Production

The research was carried out on a fermented food product (FFP) including soya flour, alfalfa and malta sprouts, along with other minor components obtained directly from the manufacturers (Pentabiol S.L, Navarre, Spain; www.pentabiol.es/?lang=en). The appearance of the FFP is similar to fine sawdust and presents a mean particle size of 0.1 mm (Figure S1). During the production of FFP the first stage covers the fermentation of a mixture of pre-cultured starter microorganisms, including LAB and yeasts, with other minor components. The second phase includes the incorporation of this culture to the raw materials for a second fermentation. At the end, air drying is used to reduce moisture content in the final product.

### 2.2. Experimental Design

Experiments were run from the product fabrication (0 month) to its best-before date (12 months) including some intermediate time points (1, 3, and 6 months). The product was packaged in two different conditions and stored at four different temperatures. The effect of oxygen exposition was tested with the utilization of two different packaging conditions, standard packaging (SP) and vacuum packaging (VP) (Figure S2). The selected storage temperatures ranged from low temperatures (freezing at −20 °C (F) and cooling at 4 °C (C)) to high temperature (37 °C (HT)). Additionally, room temperature (RT) was set with a portable measuring instrument (Humidity/Temperature Data Logger PCE-HT 71N, PCE, Spain). Table 1 summarizes the experimental conditions employed and the samples coding. RT and SP were used as temperature and packaging reference conditions, respectively. 

### 2.3. Sample Preparation

Freshly produced FFP was portioned and bagged in individual packages containing 150 g of the product. Each experimental condition was replicated twice (and performed repeated measures) and individual bags were created for the measurement of each microbiological and physicochemical parameter to facilitate experiment execution. In order to mimic as close as possible regular sacks commercialized by the manufacturer, the same package (a three layer bag containing two paper layers and a plastic layer in between) and sealing technique (industrial sack sewing machine) was employed. Vacuum packaging was performed using polyethylene plastic bags and a vacuum sealer (Silver Crest, Hamburg, Germany). The final number of required bags was 256 (eight conditions × two duplicates × three parameters × four time points). With the purpose to ensure that we had the necessary samples, some extra packs were prepared and exposed to all the experimental conditions. See the experimental design scheme in Figure S3.

Before any test, all samples were adjusted to RT. Prior to every experiment, the content of the package was mixed thoroughly using a sterile spatula and the sample was analyzed according to the different protocols. During sample handling gloves were used and working areas were sterilized with 70% alcohol. Contamination was avoided using gas burners.

### 2.4. Microbiological Analysis 

Viable bacteria were determined by classical culture-based methods at each sampling time (0, 1, 3, 6, and 12 months). The amount of total aerobic bacteria (total bacteria), LAB, and yeasts was determined by using Plate Count Agar (PCA) (Sigma), de Man, Rogosa, and Sharpe agar (MRS) (Sigma), and Sabouraud Glucose agar with chloramphenicol (Sigma) mediums, respectively. All media were prepared following manufacturer’s instructions, autoclaved at 120 °C for 15 min and cooled to 42–45 °C before use. For every sample a 1:10 dilution (extract) was prepared with 10 g of FFP and 90 mL of 0.85% sterile saline solution containing 0.1% of peptone from casein (Scharlau, Sentmenat, Spain). The mixture was poured in a sterile stomacher bag and homogenized for 2 min with a Stomacher (LB400 Homogenizer, VRW International). The resultant product was then transferred to a sterile glass bottle through the stomacher bag filter and serial 10-fold dilutions in sterile saline solution were prepared. All plates were inoculated by standard pour plate method (1 mL of sample solution and 20 mL of medium) except for MRS agar, which was cultured by spread plate method (100 μL of sample solution in 20 mL of solid medium), as recommended by the European Standard EN 15787:2009 for the isolation and enumeration of *Lactobacillus* spp. in animal feeding stuffs. All dilutions were plated in duplicate and two negative control plates were prepared for each medium. MRS plates were grown in the culture conditions referenced above (anaerobic incubation at 37 °C for 72 h). PCA and Sabouraud plates were incubated as indicated by the European Standard EN ISO 4833-1:2013 (aerobic incubation at 30 ± 1 °C for 72 ± 3 h) and ISO 7954:1987 (aerobic incubation at 22–25 °C for 3–5 days), respectively. After the incubation period plates were counted and the average number of colony forming units (CFU) per gram of FFP was calculated. Data is presented as mean of duplicate determinations (plating) from a single extract. Plates containing less than 4 CFU were counted as <10 CFU/g of sample. 

### 2.5. Physicochemical Analysis

The pH was measured at RT by electrode immersion with a pH meter Crison Model 2001 (Crison Instrument S.A., Barcelona, Spain). A solution with 10 g of the FFP and 90 mL of sterile deionized water was prepared in duplicate for each replica. Measurements were performed in triplicate in agitation with a magnetic stirrer to avoid sample sedimentation. 

For the determination of the moisture content (MC) and according to the referenced international method available for cereals and cereals products (ISO 712:2009), 5 ± 1 g of sample was used and left to dry at 130 °C for 2 h. Measurements were performed in duplicate for each replica. The percentage of water present in the sample was calculated using the given formula *MC*% = (*m*_0_ − *m*_1_/*m*_0_) × 100, where *m*_0_ refers to the initial mass and *m*_1_ refers to the mass after drying. 

### 2.6. Statistical Analysis

All statistical procedures were performed using SPSS software for Microsoft (IBM SPSS Statistics 20). 

Data from each sampling time (1, 3, 6, and 12 months) and parameter (total bacteria, LAB, yeasts, pH, and MC) were submitted to univariate analysis of variance (ANOVA) by using the generalized linear model (GLM). Comparisons were performed between the different categories of temperature and packaging and the reference conditions: RT and SP, respectively. The significance level was set to *p* < 0.05, and *p* < 0.01 and *p* < 0.001 were considered highly significant and extremely significant, respectively. Data are presented as mean ± standard deviation (SD).

The Spearman correlation analysis was performed and Spearman correlation coefficient (ρ) was estimated to determine the linear association between the following variables pH, MC, total bacteria, LAB, and yeasts (n = 80). The outcome results were interpreted according to the degree of association as very high (ρ = 0.9–1), high (ρ = 0.7–0.9), moderate (ρ = 0.5–0.7), or low (ρ = 0.2–0.5) after taking significant correlation (*p* < 0.05) values into consideration.

## 3. Results

### 3.1. Dynamics of Total Bacteria and LAB Stored under Different Temperature and Packaging Conditions

The results for the effects of storage temperature and packaging mode on the counting of total bacteria in FFP are shown in Figure S4. Overall, FFP experimented a reduction in the load of total bacteria after 12 months of storage, that fluctuated between 8% and 44% in C and HT, respectively. F and RT had intermediate values (9% and 26%, respectively). Undoubtedly, F and C temperature were the conditions that preserved better the content of total bacteria in FFP, which experienced a reduction of only 0.47 and 0.40 log units, respectively, after one year of storage. On the contrary, HT presents the more challenging temperature condition for total bacteria because up to 2.09 log units were lost during the same period.

**Figure 1:**
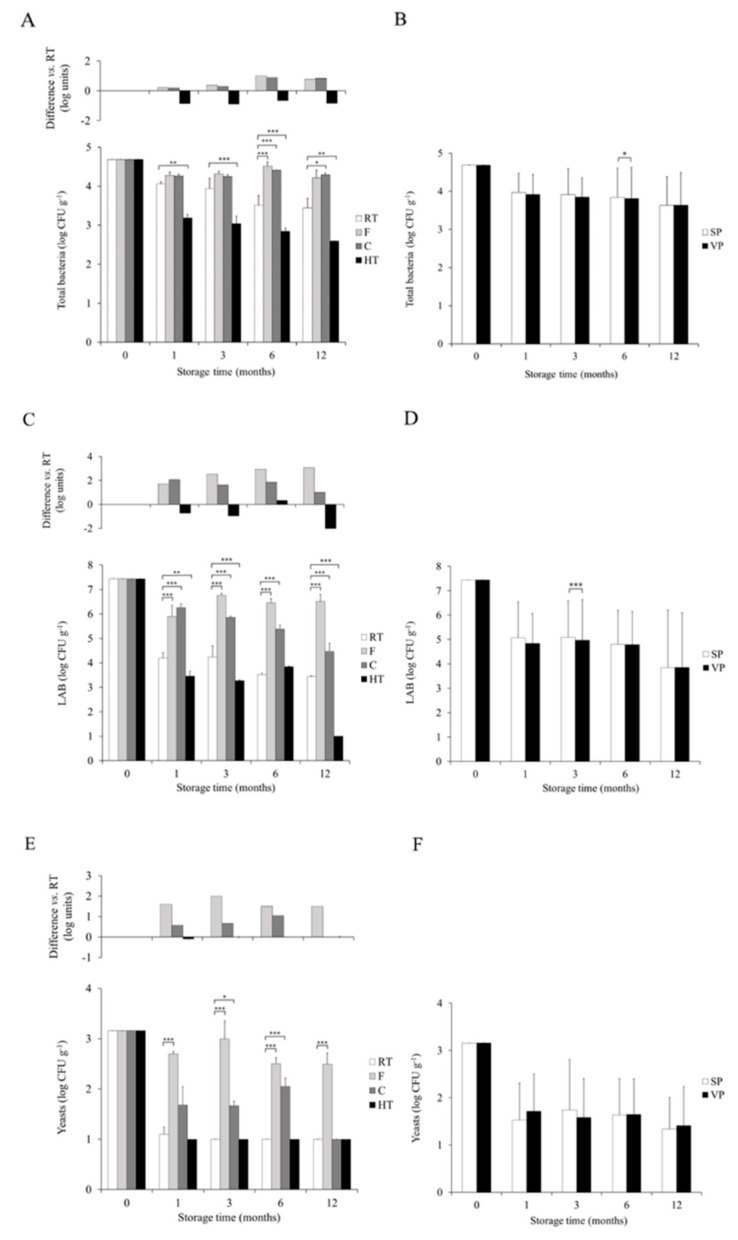
Effect of storage temperature and packaging mode on the counts of total bacteria (log colony forming units (CFU) g^−1^) (**A**,**B**), lactic acid bacteria (LAB) (log CFU g^−1^) (**C**,**D**), and yeasts (log CFU g^−1^) (**E**,**F**) in fermented food product (FFP) samples. RT: room temperature; F: freezing; C: cooling; HT: high temperature; SP: standard packaging; VP: vacuum packaging. * *p* < 0.05, ** *p* < 0.01, *** *p* < 0.001.

When the effect of storage temperature was compared between the temperature conditions some significant differences were also found (Figure 1A). During the first 3 months the number of total bacteria in C and F temperature was comparable to that in RT (*p* > 0.05 at 1 and 3 months). At 6 months, however, C and F temperature had greater number of total bacteria than RT (*p* < 0.001 and *p* < 0.001 in F and C, respectively). At 12 months significance was only observed in C temperature (*p* < 0.05). The number of total bacteria in HT was smaller than RT in all the sampling points (*p* < 0.01, *p* < 0.001, *p* < 0.001, and *p* < 0.01 at 1, 3, 6, and 12 months, respectively). 

In regard to packaging, total bacteria count in FFP was similar in SP and VP at all the sampling times, and statistical significance (*p* < 0.05) was found only at 6 months, the total bacteria load being lower in VP (Figure 1B).

Concerning viable LAB in FFP, some differences were found among the studied experimental conditions too (Figure S5). Baseline LAB load experienced a sharp decline after 12 months, with the exception of F temperature. At 12 months, samples at RT lost half of viable LAB content (53% of loss), samples stored at lower temperature (F and C) showed a slighter decline (12% and 39% of loss, respectively) while samples stored at HT suffered the greatest viability decrease (86%). Samples stored at F temperature only lost 0.93 log units. Such decrease is small in comparison with the drops of 2.98, 4, and 6.44 log units found in C, RT, and HT, respectively. Indeed, samples at HT got the lowest LAB load at 12 months with <1 log CFU/g, while the other conditions managed to keep values over 3.44 log CFU/g at that time.

Comparison of the survival of LAB between RT and the other temperature conditions demonstrated statistically significant differences at all time points analyzed (Figure 1C). In F and C temperatures the number of LAB was statistically significantly higher (*p* < 0.001) than in RT at 1, 3, 6, and 12 months. Indeed, at 12 months the counts of LAB in F temperature were high and considerably greater than the load found in the remaining temperature conditions, including C temperature. In the case of LAB in FFP, F condition is the most favorable one. On the other hand, HT had lower LAB counts than RT (*p* < 0.001) at 1, 3, and 12 months. 

The packaging mode only had a subtle effect on LAB and statistically significant differences between SP and VP were only identified at 3 months (*p* < 0.001), the time in which SP presented 0.13 log CFU/g more than VP (Figure 1D).

### 3.2. Dynamics of Yeasts Stored under Different Temperature and Packaging Conditions

The obtained average values of yeasts are given in Table S6. Following 1 month of storage, the load of yeasts drastically declined in all the temperature conditions (2.16 log units in RT and HT, 1.94 log units in C) excluding F temperature (0.46 log units). Similarly, at 12 months C, RT, and HT had lost 2.16 log units and F had only lost 0.67 log units. These results account for 67% and 20% of loss, respectively.

Yeasts displayed some slightly different dynamics when FFP was exposed to different storage temperature (Figure 1E). RT and HT had a comparable effect on yeast survival and no statistically significant differences were found at any time. On the other hand, relevant differences between storage at RT and low temperature conditions were identified. F temperature led to higher (*p* < 0.001) counts of yeasts at all the sampling times. For C temperature, no statistically significant differences were found at 1 month (*p* = 0.05), however, significantly lower values were found at 3 (*p* < 0.05) and 6 months (*p* < 0.001). At the end of the study only the F temperature differed from RT in yeast content.

Focusing on the packaging mode, VP did not provoke differences in viability of yeasts in FFP (Figure 1F). 

### 3.3. The Influence of Temperature Conditions and Packaging Modes on pH 

Values of pH measurements are summarized in Table S7. During the study and at the end of the study (12 months), the pH in all temperature and packaging conditions remained almost invariable in comparison to the initial pH value. 

**Figure 2:**
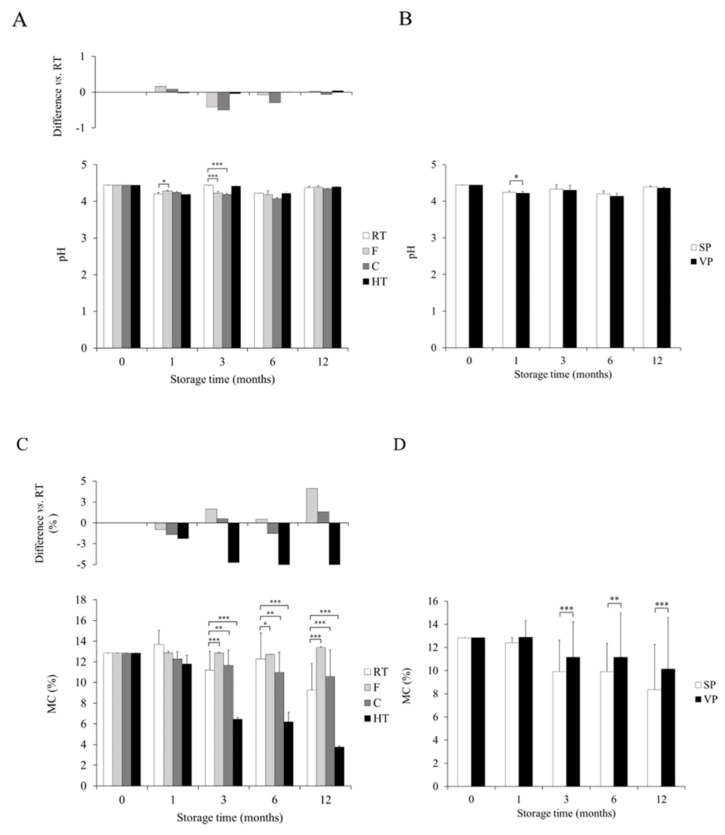
Effect of storage temperature and packaging mode on pH (**A**,**B**) and moisture content (MC) (%) (**C**,**D**) in fermented food product (FFP) samples. RT: room temperature; F: freezing; C: cooling; HT: high temperature; SP: standard packaging; VP: vacuum packaging. * *p* < 0.05, ** *p* < 0.01, *** *p* < 0.001.

Concerning the storage temperature, only some differences were observed between FFP stored at RT and at low temperature (Figure 2A). pH in F and RT was comparable in all the sampling times except 3 months, where a decrease was observed (*p* < 0.001) in the former condition. In the case of C temperature significant differences with RT were observed at 1 and 3 months, being lower (*p* < 0.05) at 1 month and greater (*p* < 0.001) at 3 months in RT vs. C temperature. Statistically significant differences were not found between HT and RT at any time.

Packaging only showed to have a significant effect on FFP’s pH values at 1 month, when VP presented a lower (*p* < 0.05) pH compared to SP (Figure 2B). 

### 3.4. The Influence of Temperature Conditions and Packaging Modes on Moisture Content

The values obtained after MC determination are shown in Table S8. The degree of MC loss in FFP varied broadly from 5% to 70% of loss at 12 months and such loss was a gradual. Remarkably, a clear effect of temperature and packaging can be concluded since MC was very different between the eight samples. 

When MC was compared between RT and the experimental conditions some differences were found at 3, 6, and 12 months (Figure 2C). F temperature was the condition which best preserved MC, and had greater values than RT from 3 months to the end of the study (*p* < 0.001 at 3 and 12 months; *p* < 0.05 at 6 months). With reference to C temperature, it showed higher MC than RT at 3 (*p* < 0.01) and 12 (*p* < 0.001) months but at 6 months the numbers were over RT values (*p* < 0.01). HT presented lower (*p* < 0.001) MC than RT at 3, 6, and 12 months. 

With respect to packaging, during the study MC behaved almost identically in both packaging modes (Figure 2D). A gradual decline in MC occurred during FFP storage. No differences were found at 1 month, however, a considerable fall was registered between 1 and 3 months, after which MC remained almost unchanged (6 months) until a tiny final decline at the end of the study. Significant differences (*p* < 0.001) were found at 3, 6, and 12 months. At all the sampling time points VP preserved MC better than SP.

### 3.5. Interplay between Physicochemical and Microbiological Profile 

Descriptive statistics of Spearman´s correlation coefficient (ρ) and the *p*-value are depicted in Table 2. Spearman´s correlation analysis revealed the statistically significant low positive correlation between pH and total bacteria (ρ = 0.228; *p* = 0.042), pH and LAB (ρ = 0.262; *p* = 0.019), and pH and yeasts (ρ = 0.293; *p* = 0.008). Similarly, a moderate positive correlation was observed between MC and total bacteria (ρ = 0.557; *p* < 0.001), MC and LAB (ρ = 0.618; *p* < 0.001), and MC and yeasts (ρ = 0.616; *p* < 0.001). Moreover, the analyzed microbiological profiles showed a high or very high positive correlation between them, total bacteria and LAB (ρ = 0.876; *p* < 0.001), total bacteria and yeasts (ρ = 0.846; *p* < 0.001), and LAB and yeasts (ρ = 0.913; *p* < 0.001).

In regard to pH and MC, a statistically significant correlation was not found between the analyzed physicochemical parameters (*p* = 0.648).

Reports of model coefficient values of total bacteria, LAB, yeasts, pH, and MC are available in Tables S1–S5.

## 4. Discussion

The main purpose of the present study was to draw attention to how storage conditions influence the microbial community present in FFP. The first variable that we considered analyzing was the load of viable microorganisms in FFP measured in specific microbiological media. Secondly, given that the nature of the food component can compromise microbial survival [23,25], the most important physicochemical parameters were also monitored and their influence on the microbial load was evaluated. Some authors had previously listed the key factors on probiotic viability [23,24,53] and with the exception of food processing, which was beyond the scope of this study, we have addressed most of them: characteristics of the food matrix, product packaging, storage condition, and microbiological profile.

In the present work we aimed to monitor the potentially beneficial bacteria load in FFP as previously determined in other food carriers [46,54,55,56]. Although the microorganisms in FFP resisted production and manufacturing and do not seem to be extremely sensitive to external agents [57], our findings revealed a reduction in the initial load. We presume that it was originated by changes in nutrient availability [29,58], exposure to products of the metabolism [56,59], and interactions within other microbial species [60,61], which can concurrently be motivated by external factors such as storage temperature, packaging, and time [44,62].

### 4.1. Bacterial Viability in FFP

As above mentioned, environmental temperature is a key regulator of microbial survival and can be deleterious for bacteria stability [22,56]. Hypothetically and in agreement with the available scientific evidence [24,63], the most suitable temperature for the survival of microorganisms in FFP would be low temperature: freezing or cooling. According to our results and focusing on total bacteria, for a short storage time (3 months or less), storage at low temperatures (F or C) does not have advantages over RT, being that both had comparable counts of total bacteria. For storage periods longer than 6 months, however, it would be better to store FFP at F or C temperature. Regarding LAB, they were more sensitive to storage than total bacteria. Following 1 month of storage low temperatures were better than RT for LAB’s survival. It appears that F is the most convenient condition, far better than C. Our findings share a number of similarities with earlier studies which reported that low temperature is helpful in preserving the microbial load [29,34,63,64]. 

### 4.2. Yeast Viability in FFP

In spite of the fact that bacteria have received the most attention as probiotic microorganisms, yeasts present an alternative or complementary source with probiotic effects [65] and contribute with a number of technological properties of substantial interest in food production [66]. In contrast to bacteria, there has been little discussion on the stability of yeasts in food products and reports on the cell counts of yeasts through storage are scarce. Clearly, storage at 37 °C or above results in detrimental viability of prokaryotic and eukaryotic microorganisms in FFP. This could be attributed to the great impact that high temperature has on the water content, which may indirectly compromise microbial viability as hypothesized by other authors [67]. 

### 4.3. Interplay between Microbial Groups

In complex mixtures of microorganisms like some fermented foods, the presence of specific microbes can modify the final balance with a beneficial or deleterious effect [8,60,61]. Some microorganisms can promote the survival of others through the liberation of growth-promoting factors to the media [31,68]. For instance, some published reports indicate that the presence of yeasts is favorable for the maintenance of LAB viability, probably because of their nutritional properties [39,69,70]. On the other hand, the combination of both LAB and yeasts may be detrimental for the latter, since some LAB-derived molecules or metabolites such as acetic acid [57] or bacteriocins [71] showed an antifungal activity [30,65]. It has also been reported that in situations in which both yeasts and bacteria coexist in the same matrix, conditions of high pH (above neutral pH) are especially damaging for the former, which suffer a decline in their growth because of the competitive advantage of bacteria [72]. Considering that, it is likely that some interactions happened between bacteria and yeasts that coexist in FFP. Our data pointed out that LAB, total bacteria, and yeasts showed a high positive correlation, so it could be speculated that there was not an inhibitory or competitive exclusion between bacteria and yeasts in FFP’s ecosystem.

### 4.4. Minor Effect of Packaging Mode on FFP’s Microorganisms

Besides environmental temperature, exposure to oxygen is another relevant parameter to take into consideration for bacterial survival and growth. Generally, oxygen has a detrimental effect on bacterial survival either directly with peroxidation reactions [24] and generation of products [59], or indirectly, by affecting adjacent cells [30]. Oxygen conditions inside the experimental packs was expected to vary between standard and vacuum packaging, and consequently influence differently on the viability of the resident commensal microbes. It is somewhat surprising, however, that our results did not reveal great differences between both packaging conditions. In all the analyzed microbiological groups (total bacteria, LAB, and yeasts), vacuum packaging did not provide an advantage over the conventional packaging mode. On one hand, it is plausible that vacuum packaging failed to maintain an anaerobic environment and residual oxygen remained in the product. This situation could be caused by the relatively high permeability of polyethylene, the material used for vacuum packaging, in comparison to other packaging materials [24,53].

On the other hand, it is also likely that the oxygen exposure between packaging conditions was different, however, it did not provoke adverse consequences on the bacteria survival, as previously reported in yogurt [73]. To confirm the role of oxygen and elucidate this issue, a study on the existing dissolved oxygen in SP and VP would be valuable. 

### 4.5. pH and Moisture Content in FFP through Storage

On the grounds that environmental conditions have a main effect on the growth kinetics of bacteria culture [29,34,39,63], we considered that the study of pH in FFP would be valuable for the understanding of what happens on the product during its storage. It is generally accepted that a decline in a pH value could be an indicator of favorable conditions for bacterial survival, as the activity of viable microorganisms can be responsible for changes in pH in the product [46], probably because of the production of organic acids [60,74]. Conversely, an extremely low pH is generally associated to a reduction in the growth yield [39] because it can lead to undissociated acids [26,30]. In FFP the load of microorganisms decreased over time, however, FFP´s pH hardly changed besides its positive correlation with total bacteria, LAB, and yeasts. It could be due to the buffering effect of the matrix, as previously reported in a beverage with milk and carrot juice inoculated with probiotics [64].

Studies on other food matrixes did observe an acidification through storage, which is hypothesized to be caused by residual microbial activity. Yogurt stored at 5 °C suffered from reductions of 0.2–0.5 units in pH and the loss was dependent on the probiotic species studied [46]. A study on cheese inoculated with probiotics revealed that pH was stable during 29 days of storage at 4 °C, however, when the storage was at 12 °C a significant acidification occurred in the samples. Again, the change was dependent on the inoculated probiotic bacteria [68]. The authors suspected that the indirect stimulation of bacteria viability by microbial metabolites may explain pH reduction. For example, in dry fermented sausages, pH significantly increased through 120 days storage under different temperatures (4, 22, and 37 °C), and the storage at 37 °C had the biggest impact on the pH [74]. Other products like boza [49] or some fermented dairy products [46] had a significant drop in pH even when stored at cooling temperature. These findings suggest that is more than likely that the nature of food ingredients governs how acidity changes through storage.

Likewise, we considered that MC could be somehow relevant for the viability of microorganisms so it was explored as another physicochemical parameter. The water content in a food matrix has a clear direct effect on the pressure of the cell walls and determines the osmotic pressure, which may be detrimental for microbial viability [34,35] and is a strong growth-limiting factor for yeasts [75]. The water content is of special interest in frozen or freeze-dried cultures [23,76,77], however, less information is available regarding how water present in a food matrix influences microbial survival. 

### 4.6. Overall Influence of Storage on FFP 

**Figure 3:**
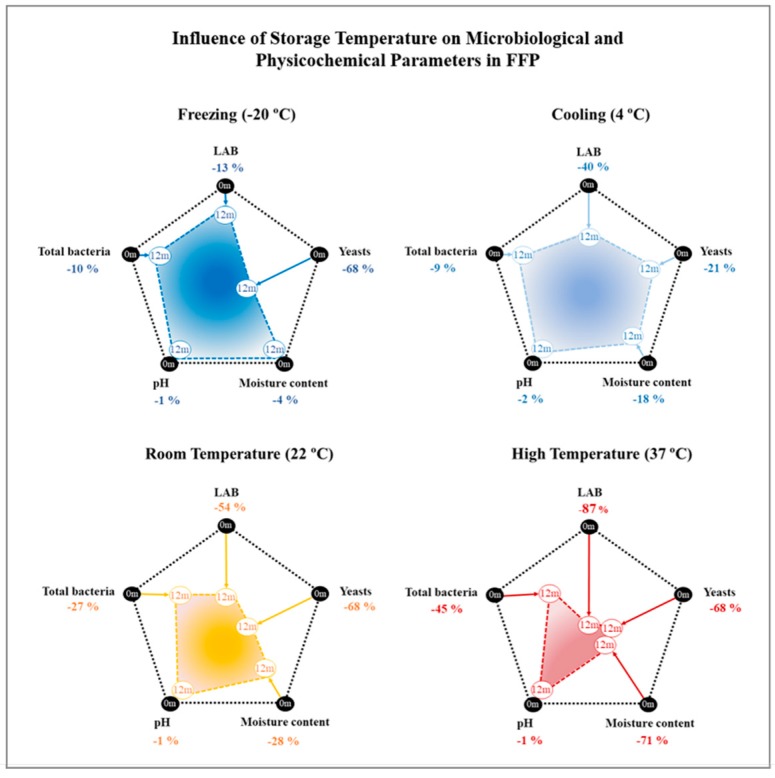
Overall influence of the analyzed temperature conditions on the microbiological profile and physicochemical properties of the fermented food product (FFP) following 12 months of storage. 0m: values obtained at the beginning of the study; 12m: values determined at the end of the study for each temperature condition. LAB: lactic acid bacteria.

Figure 3 summarizes the overall influence of storage temperature on total bacteria, LAB, yeasts, pH, and moisture content in FFP samples following 12 months of storage. As noted above, temperature had a considerably greater impact on FFP than packaging mode. The analysis performed suggests that high temperature had a greater effect on all the analyzed parameters, while lower temperature preserved baseline values better. The adverse effects of high temperature on the survival of the alive microorganisms seems to be proportional to the storage time. Even though food distribution normally takes a few months, preventive actions should be taken to ensure that transport, shipping, and manipulation of FFP do not expose the product to high temperature. Moreover, when possible, cold chain must be set in order to impact as little as possible the alive microorganisms present in FFP. 

To conclude, we can propose the optimal storage conditions for FFP according to the results obtained. On the grounds that LAB present interesting beneficial effects on the host [6,78] it would be advised to prioritize the survival of LAB over other bacteria groups. Hence, the storage of FFP at F or C temperature as long as possible would be recommended.

Besides, in cases where storage at low temperature is not feasible, it would be advisable to store FFP protected from the light exposure and to consume it in a period of time that does not exceed 3 months. Regarding packaging, vacuum packaging did not show a protective effect on bacteria and yeast survival. Therefore, for the storage of FFP standard packaging would be as useful as vacuum packaging.

## 5. Conclusions

In summary, our findings showed that some procedures may be helpful in protecting the viability of FFP’s microbiota, though the load of bacteria and yeast decreased through storage. Specifically, in relation to the storage temperature, storage at −20 and 4 °C were the most convenient conditions and therefore would be recommended. Besides, taking the results into consideration, not exceeding a period of 3 months to preserve a substantial number of viable microorganisms would be recommended. Regarding the packaging methods, vacuum packaging revealed to not be better than standard packaging.

This work has led us to conclude that FFP is a relatively stable fermented food product for livestock which could be a suitable matrix for probiotics. Therefore, FFP and other plant-based fermented products with similar characteristics may be useful as novel probiotic delivery systems. 

It should be noted that the present research was only an attempt to understand the dynamics of the complex microbial ecosystem in the FFP matrix. Given the clinical and technological relevance of bacteria identification up to strain level and the characterization of bioactive metabolites in foods, future studies with genomic and metabolomic approaches should be conducted to deepen understanding of the dynamics that take place in the FFP matrix.

## Figures and Tables

**Table 1:** Experimental conditions and sample coding.

Experimental Conditions		Sample Code
Storage Temperature	Packaging Mode	
Freezing (−20 °C)	Standard	F-SP
	Vacuum	F-VP
Cooling (4 °C)	Standard	C-SP
	Vacuum	C-VP
Room temperature (22 °C) *	Standard	RT-SP
	Vacuum	RT-VP
High temperature (37 °C)	Standard	HT-SP
	Vacuum	HT-VP

* Data from the Humidity/Temperature Data Logger revealed that the temperature in the laboratory was 21.81 ± 2.2 °C, so RT was set at 22 °C. F-SP: freezing standard packaging; F-VP: freezing vacuum packaging; C-SP: cooling standard packaging; C-VP: cooling vacuum packaging; RT-SP: room temperature standard packaging; RT-VP: room temperature vacuum packaging; HT-SP: high temperature standard packaging; HT-VP: high temperature vacuum packaging.

**Table 2:** Spearman´s correlation coefficient (ρ) and its level of significance (*p*-value) for the analyzed physicochemical and microbiological parameters.

	pH		MC		Total Bacteria		LAB		Yeasts	
	ρ	*p-*Value	ρ	*p-*Value	ρ	*p-*Value	ρ	*p-*Value	ρ	*p-*Value
**pH**			0.052	0.648	0.228	0.042	0.262	0.019	0.293	0.008
**MC**	0.052	0.648			0.557	<0.001	0.618	<0.001	0.616	<0.001
**Total bacteria**	0.228	0.042	0.557	<0.001			0.876	<0.001	0.846	<0.001
**LAB**	0.262	0.019	0.618	<0.001	0.876	<0.001			0.913	<0.001
**Yeasts**	0.293	0.008	0.616	<0.001	0.846	<0.001	0.913	<0.001		

MC: moisture content; LAB: lactic acid bacteria.

## Supplementary Materials

The following are available online at https://www.mdpi.com/2304-8158/9/3/302/s1, Figure S1: Sample of fermented food product, Figure S2: Different packaging modes used in the study, Figure S3: Experimental design scheme, Figure S4: Counts of total bacteria (log CFU g^−1^) in FFP samples stored under different storage temperatures and packaging modes, Figure S5: Counts of LAB (log CFU g^−1^) in FFP samples stored under different storage temperatures and packaging modes, Figure S6: Counts of yeast (log CFU g^−1^) in FFP samples stored under different storage temperatures and packaging modes, Figure S7: pH in FFP samples stored under different storage temperatures and packaging modes, Figure S8: Moisture content (%) in FFP samples stored under different storage temperatures and packaging modes, Table S1: Report of model coefficient values, confidence intervals and *p*-values of the total bacteria generalized linear model analysis (GLM), Table S2: Report of model coefficient values, confidence intervals, and *p*-values of LAB generalized linear model analysis (GLM), Table S3: Report of model coefficient values, confidence intervals, and *p*-values of the yeast generalized linear model analysis (GLM), Table S4: Report of model coefficient values, confidence intervals, and *p-*values of the pH generalized linear model analysis (GLM), Table S5: Report of model coefficient values, confidence intervals, and *p*-values of the moisture content (%) generalized linear model analysis (GLM).
