# Supplementary material for: Influence of Storage Temperature and Packaging on Bacteria and Yeast Viability in a Plant-Based Fermented Food
Source: Foods. 2020 Mar 7;9(3):302. doi: 10.3390/foods9030302 (PMC7143826; doi:10.3390/foods9030302)
Supplement: Supplementary file 1 [file foods-09-00302-s001.zip › Supplementary materials/Supplementary materials 1.pdf]

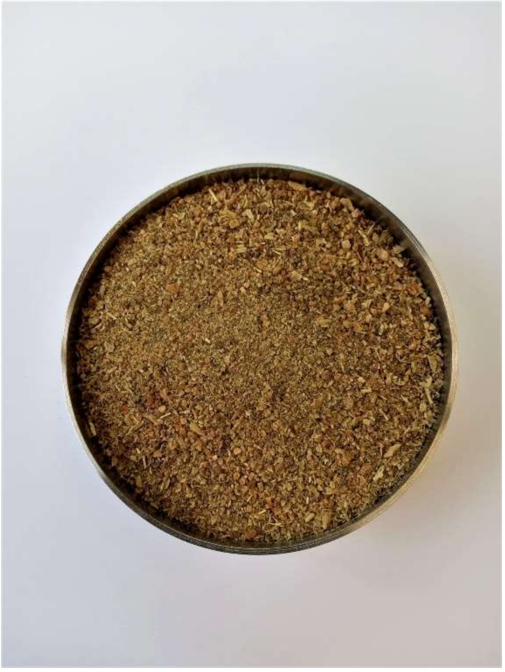

Figure S1. Sample of fermented food product (FFP).

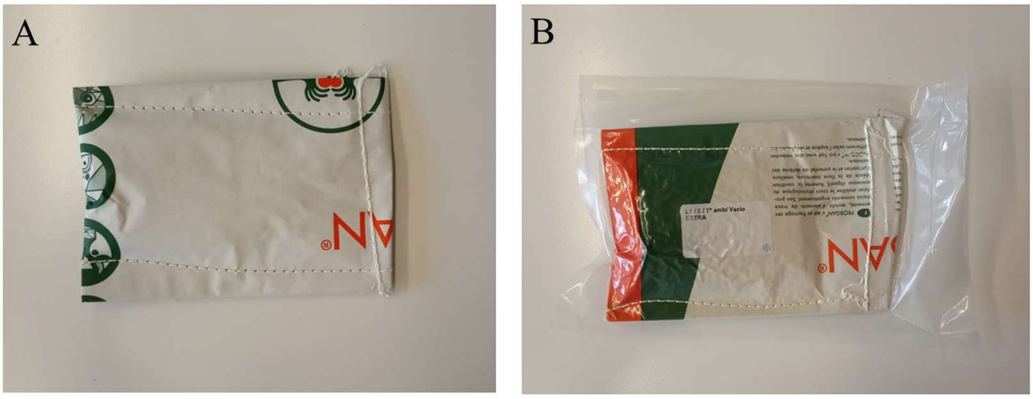

Figure S2. Different packaging modes used in the study. Standard packaging (**A**) and vacuum packaging (**B**).

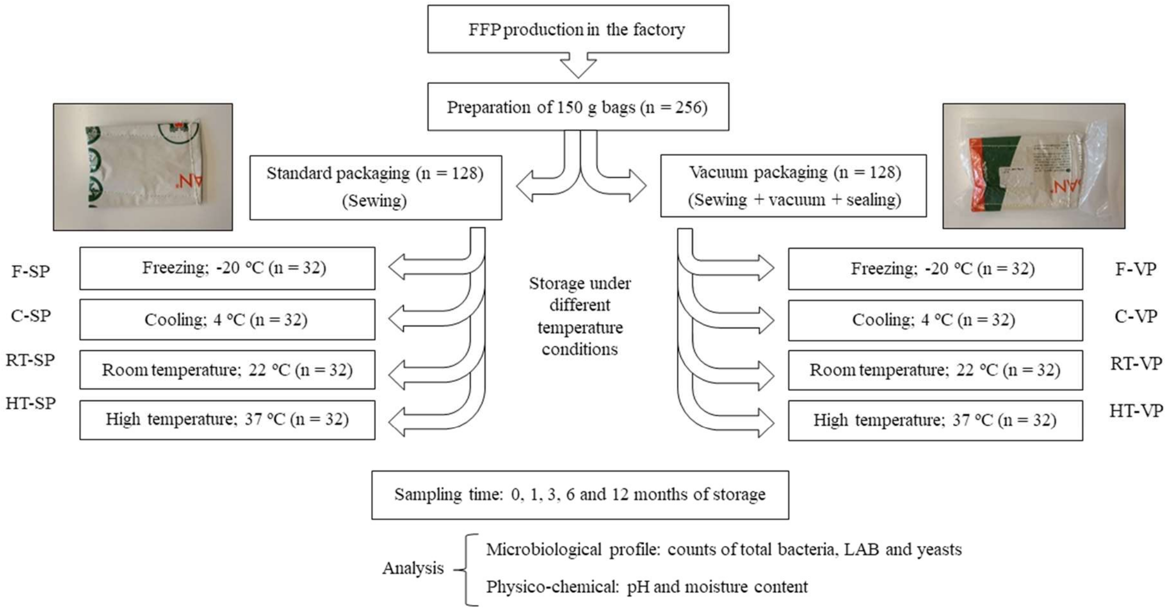

Figure S3. Experimental design scheme. FFP: fermented food product; LAB: lactic acid bacteria; F-SP: freezing standard packaging; C-SP: cooling standard packaging; RT-SP: room temperature standard packaging; HT-SP: high temperature standard packaging; C-VP: cooling vacuum packaging; RT-VP: room temperature vacuum packaging; HT-VP: high temperature vacuum packaging.

Table S1. Report of model coefficient values, confidence intervals and *p*-values of the total bacteria generalized linear model analysis (GLM).

Parameter	Storage time (months)	Condition	β	95% CI		*p*-value
Temperature	1	F	0.315	-0.049	0.679	0.081
		C	0.270	-0.094	0.634	0.126
		HT	-0.770	-1.134	-0.406	<0.01
	3	F	0.225	-0.242	0.692	0.299
		C	0.150	-0.317	0.617	0.480
		HT	-1.220	-1.687	-0.753	<0.001
	6	F	0.750	0.477	1.023	<0.001
		C	0.725	0.452	0.998	<0.001
		HT	-0.900	-1.173	-0.627	<0.001
	12	F	0.455	-0.126	1.036	0.180
		C	0.635	0.054	1.216	0.036
		HT	-1.020	-1.601	-0.439	<0.01
Packaging	1	VP	0.075	-0.289	0.439	0.647
	3		-0.380	-0.847	0.087	0.097
	6		-0.350	-0.623	-0.077	0.018
	12		-0.355	-0.936	0.226	0.196

Comparisons were computed between the different conditions of temperature and packaging and its reference condition, RT and SP respectively. CI: confidence interval; F: freezing; C: cooling; HT: high temperature; VP: vacuum packaging; RT: room temperature; SP: standard packaging.

Table S2. Report of model coefficient values, confidence intervals and *p*-values of LAB generalized linear model analysis (GLM).

Parameter	Storage time (months)	Condition	β	95% CI		*p*-value
Temperature	1	F	1.875	1.397	2.353	<0.001
		C	2.025	1.547	2.503	<0.001
		HT	-1.025	-1.503	-0.547	<0.01
	3	F	2.140	1.912	2.368	<0.001
		C	1.275	1.047	1.503	<0.001
		HT	-1.255	-1.483	-1.027	<0.001
	6	F	3.015	2.458	3.572	<0.001
		C	1.685	1.128	2.242	<0.001
		HT	0.255	-0.302	0.812	0.322
	12	F	3.260	3.002	3.518	<0.001
		C	0.760	0.502	1.018	<0.001
		HT	-2.455	-2.713	-2.197	<0.001
Packaging	1	VP	-0.315	-0.793	0.163	0.167
	3		-0.645	-0.873	-0.417	<0.001
	6		-0.095	-0.652	0.462	0.704
	12		-0.035	-0.293	0.223	0.763

Comparisons were computed between the different conditions of temperature and packaging and its reference condition, RT and SP respectively. LAB: lactic acid bacteria; CI: confidence interval; F: freezing; C: cooling; HT: high temperature; VP: vacuum packaging; RT: room temperature; SP: standard packaging.

Table S3. Report of model coefficient values, confidence intervals and *p*-values of the yeasts generalized linear model analysis (GLM).

Parameter	Storage time (months)	Condition	β	95% CI		*p*-value
Temperature	1	F	1.670	1.255	2.085	<0.001
		C	0.415	0.000	0.830	0.050
		HT	-1.11e-16	-0.415	0.415	1.000
	3	F	2.245	1.671	2.819	<0.001
		C	0.725	0.151	1.299	0.019
		HT	-7.307e-17	-0.574	0.574	1.000
	6	F	1.590	1.394	1.786	<0.001
		C	0.930	0.734	1.126	<0.001
		HT	8.568e-16	-0.196	0.196	1.000
	12	F	1.340	1.041	1.639	<0.001
		C	-4.935e-16	-0.299	0.299	1.000
		HT	-4.935e-16	-0.299	0.299	1.000
Packaging	1	VP	0.200	-0.215	0.615	0.298
	3		-1.388e-17	-0.574	0.574	1.000
	6		8.327e-16	-0.196	0.196	1.000
	12		-8.755e-16	-0.299	0.299	1.000

Comparisons were computed between the different conditions of temperature and packaging and its reference condition, RT and SP respectively. CI: confidence interval; F: freezing; C: cooling; HT: high temperature; VP: vacuum packaging; RT: room temperature; SP: standard packaging.

Table S4. Report of model coefficient values, confidence intervals and *p*-values of the pH generalized linear model analysis (GLM).

Parameter	Storage time (months)	Condition	β	95% CI		*p*-value
Temperature	1	F	0.060	0.019	0.101	0.010
		C	0.035	-0.006	0.076	0.086
		HT	-0.035	-0.076	0.006	0.086
	3	F	-0.185	-0.230	-0.140	<0.001
		C	-0.235	-0.280	-0.190	<0.001
		HT	-0.010	-0.055	0.035	0.622
	6	F	0.045	-0.285	0.375	0.762
		C	-0.125	-0.455	0.205	0.408
		HT	0.030	-0.300	0.360	0.839
	12	F	0.010	-0.058	0.078	0.743
		C	-0.055	-0.123	0.013	0.099
		HT	-5.329e-15	-0.068	0.068	1.000
Packaging	1	VP	-0.045	-0.086	-0.004	0.036
	3		-0.005	-0.050	0.040	0.804
	6		0.000	-0.330	0.330	1.000
	12		-0.050	-0.118	0.018	0.128

Comparisons were computed between the different conditions of temperature and packaging and its reference condition, RT and SP respectively. CI: confidence interval; F: freezing; C: cooling; HT: high temperature; VP: vacuum packaging; RT: room temperature; SP: standard packaging.

Table S5. Report of model coefficient values, confidence intervals and *p*-values of the moisture content (%) generalized linear model analysis (GLM).

Parameter	Storage time (months)	Condition	β	95% CI		*p*-value
Temperature	1	F	0.035	-1.939	2.009	0.968
		C	-0.930	-2.904	1.044	0.309
		HT	-0.320	-2.294	1.654	0.718
	3	F	2.940	2.474	3.406	<0.001
		C	0.780	0.314	1.246	<0.01
		HT	-3.600	-4.066	-3.134	<0.001
	6	F	1.255	0.144	2.366	0.031
		C	-1.855	-2.966	-0.744	<0.01
		HT	-4.620	-5.731	-3.509	<0.001
	12	F	5.865	5.495	6.235	<0.001
		C	1.345	0.975	1.715	<0.001
		HT	-3.620	-3.990	-3.250	<0.001
Packaging	1	VP	1.925	-0.049	3.899	0.055
	3		2.605	2.139	3.071	<0.001
	6		2.570	1.459	3.681	<0.01
	12		3.630	3.260	4.000	<0.001

Comparisons were computed between the different conditions of temperature and packaging and its reference condition, RT and SP respectively. CI: confidence interval; F: freezing; C: cooling; HT: high temperature; VP: vacuum packaging; RT: room temperature; SP: standard packaging.
