# Supplementary material for: Influence of Storage Temperature and Packaging on Bacteria and Yeast Viability in a Plant-Based Fermented Food
Source: Foods. 2020 Mar 7;9(3):302. doi: 10.3390/foods9030302 (PMC7143826; doi:10.3390/foods9030302)
Supplement: Supplementary file 1 [file foods-09-00302-s001.zip › Supplementary materials/Supplementary materials 2.pdf]

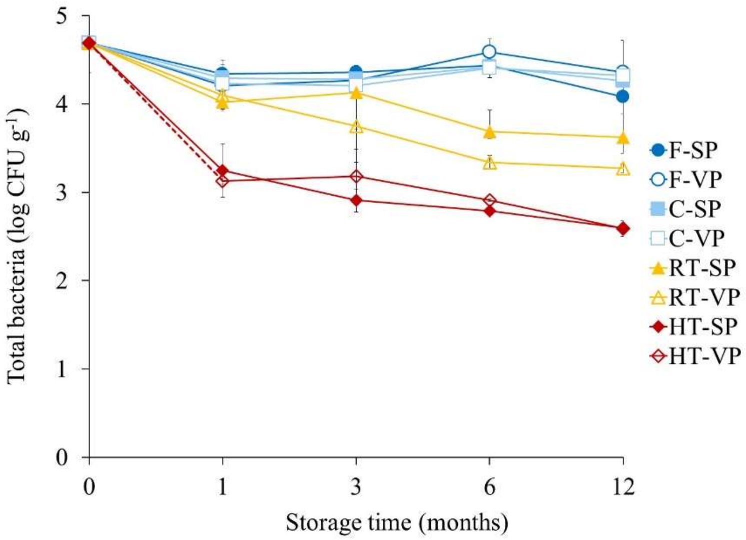


| **Sample Code** | **Temperature** | **Packaging** | **Storage time (months)** | | | | |
|---|---|---|---|---|---|---|---|
| | | | 0 | 1 | 3 | 6 | 12 |
| **F-SP** | -20 ºC | Standard | 4.69 ± 0.34 | 4.34 ± 0.11 | 4.36 ± 0.08 | 4.44 ± 0.14 | 4.08 ± 0.64 |
| **F-VP** | | Vacuum | 4.69 ± 0.34 | 4.21 ± 0.29 | 4.27 ± 0.01 | 4.59 ± 0.15 | 4.36 ± 0.03 |
| **C-SP** | 4 ºC | Standard | 4.69 ± 0.34 | 4.29 ± 0.04 | 4.28 ± 0.03 | 4.42 ± 0.02 | 4.26 ± 0.04 |
| **C-VP** | | Vacuum | 4.69 ± 0.34 | 4.23 ± 0.00 | 4.21 ± 0.08 | 4.41 ± 0.06 | 4.32 ± 0.08 |
| **RT-SP** | 22 ºC | Standard | 4.69 ± 0.34 | 4.02 ± 0.08 | 4.13 ± 0.18 | 3.69 ± 0.24 | 3.62 ± 0.27 |
| **RT-VP** | | Vacuum | 4.69 ± 0.34 | 4.10 ± 0.08 | 3.75 ± 0.41 | 3.34 ± 0.08 | 3.27 ± 0.05 |
| **HT-SP** | 37 ºC | Standard | 4.69 ± 0.34 | 3.25 ± 0.30 | 2.91 ± 0.13 | 2.79 ± 0.01 | 2.60 ± 0.03 |
| **HT-VP** | | Vacuum | 4.69 ± 0.34 | 3.13 ± 0.03 | 3.18 ± 0.31 | 2.91 ± 0.01 | 2.59 ± 0.09 |

**Figure S4.** Counts of total bacteria (log CFU $g^{-1}$) in FFP samples stored under different storage temperatures and packaging modes. FFP: fermented food product; F-SP: freezing standard packaging; F-VP: freezing vacuum packaging; C-SP: cooling standard packaging; C-VP: cooling vacuum packaging; RT-SP: room temperature standard packaging; RT-VP: room temperature vacuum packaging; HT-SP: high temperature standard packaging; HT-VP: high temperature vacuum packaging. Values are expressed as mean ± SD.

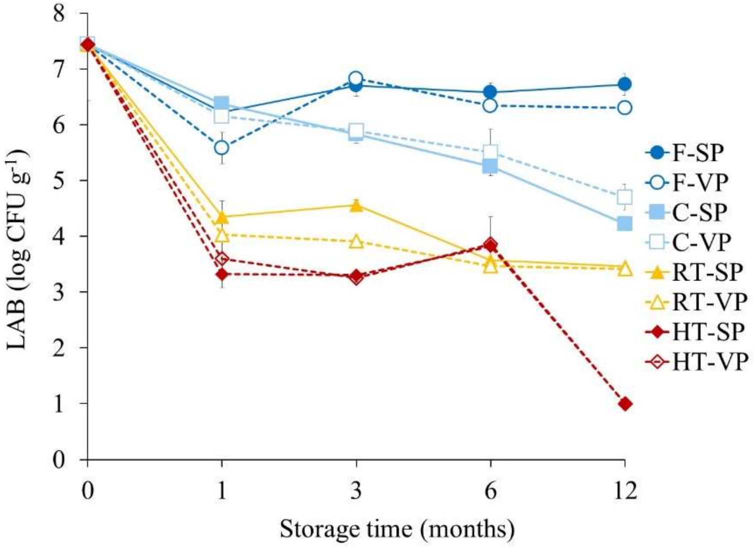


| **Sample Code** | **Temperature** | **Packaging** | **Storage time (months)** | | | | |
|---|---|---|---|---|---|---|---|
| | | | 0 | 1 | 3 | 6 | 12 |
| **F-SP** | -20 ºC | Standard | 7.44 ± 1.01 | 6.22 ± 0.06 | 6.70 ± 0.19 | 6.58 ± 0.17 | 6.72 ± 0.19 |
| **F-VP** | | Vacuum | 7.44 ± 1.01 | 5.58 ± 0.28 | 6.82 ± 0.05 | 6.34 ± 0.03 | 6.30 ± 0.03 |
| **C-SP** | 4 ºC | Standard | 7.44 ± 1.01 | 6.37 ± 0.01 | 5.83 ± 0.16 | 5.25 ± 0.10 | 4.22 ± 0.09 |
| **C-VP** | | Vacuum | 7.44 ± 1.01 | 6.15 ± 0.00 | 5.89 ± 0.00 | 5.50 ± 0.42 | 4.70 ± 0.23 |
| **RT-SP** | 22 ºC | Standard | 7.44 ± 1.01 | 4.35 ± 0.28 | 4.56 ± 0.09 | 3.57 ± 0.04 | 3.46 ± 0.01 |
| **RT-VP** | | Vacuum | 7.44 ± 1.01 | 4.03 ± 0.35 | 3.91 ± 0.03 | 3.47 ± 0.08 | 3.42 ± 0.01 |
| **HT-SP** | 37 ºC | Standard | 7.44 ± 1.01 | 3.32 ± 0.24 | 3.30 ± 0.03 | 3.82 ± 0.08 | 1.00 ± 0.00 |
| **HT-VP** | | Vacuum | 7.44 ± 1.01 | 3.60 ± 0.04 | 3.25 ± 0.07 | 3.86 ± 0.49 | 1.00 ± 0.00 |

**Figure S5.** Counts of LAB (log CFU $g^{-1}$) in FFP samples stored under different storage temperatures and packaging modes. LAB: lactic acid bacteria; FFP: fermented food product; F-SP: freezing standard packaging; F-VP: freezing vacuum packaging; C-SP: cooling standard packaging; C-VP: cooling vacuum packaging; RT-SP: room temperature standard packaging; RT-VP: room temperature vacuum packaging; HT-SP: high temperature standard packaging; HT-VP: high temperature vacuum packaging. Values are expressed as mean ± SD.

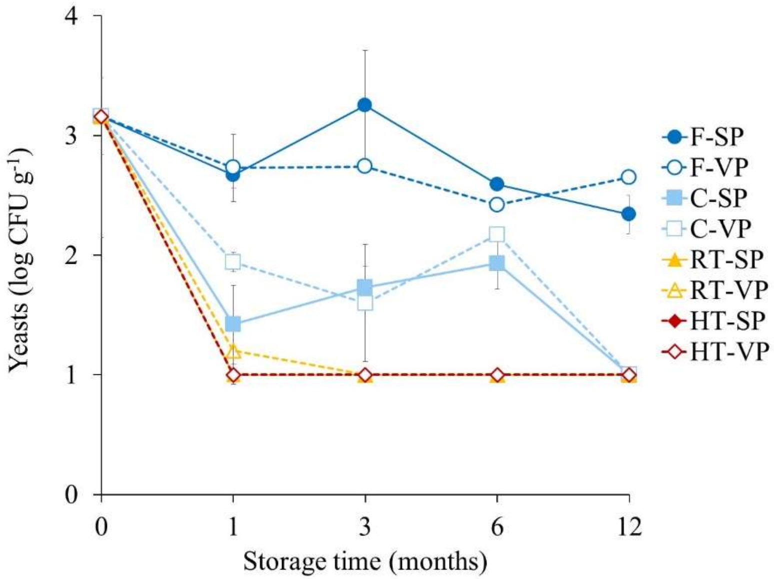


| **Sample Code** | **Temperature** | **Packaging** | **Storage time (months)** | | | | |
|---|---|---|---|---|---|---|---|
| | | | 0 | 1 | 3 | 6 | 12 |
| **F-SP** | -20 ºC | Standard | 3.16 ± 0.32 | 2.67 ± 0.11 | 3.25 ± 0.46 | 2.57 ± 0.04 | 2.34 ± 0.16 |
| **F-VP** | | Vacuum | 3.16 ± 0.32 | 2.73 ± 0.22 | 2.74 ± 0.12 | 2.39 ± 0.09 | 2.65 ± 0.33 |
| **C-SP** | 4 ºC | Standard | 3.16 ± 0.32 | 1.00 ± 0.00 | 1.43 ± 0.60 | 1.00 ± 0.00 | 1.00 ± 0.00 |
| **C-VP** | | Vacuum | 3.16 ± 0.32 | 1.44 ± 0.62 | 2.90 ± 0.20 | 1.48 ± 0.67 | 1.00 ± 0.00 |
| **RT-SP** | 22 ºC | Standard | 3.16 ± 0.32 | 1.00 ± 0.00 | 1.00 ± 0.00 | 1.00 ± 0.00 | 1.00 ± 0.00 |
| **RT-VP** | | Vacuum | 3.16 ± 0.32 | 1.00 ± 0.00 | 1.00 ± 0.00 | 1.00 ± 0.00 | 1.00 ± 0.00 |
| **HT-SP** | 37 ºC | Standard | 3.16 ± 0.32 | 1.00 ± 0.00 | 1.00 ± 0.00 | 1.00 ± 0.00 | 1.00 ± 0.00 |
| **HT-VP** | | Vacuum | 3.16 ± 0.32 | 1.00 ± 0.00 | 1.00 ± 0.00 | 1.00 ± 0.00 | 1.00 ± 0.00 |

**Figure S6.** Counts of yeasts (log CFU $g^{-1}$) in FFP samples stored under different storage temperatures and packaging modes. FFP: fermented food product; F-SP: freezing standard packaging; F-VP: freezing vacuum packaging; C-SP: cooling standard packaging; C-VP: cooling vacuum packaging; RT-SP: room temperature standard packaging; RT-VP: room temperature vacuum packaging; HT-SP: high temperature standard packaging; HT-VP: high temperature vacuum packaging. Values are expressed as mean ± SD.

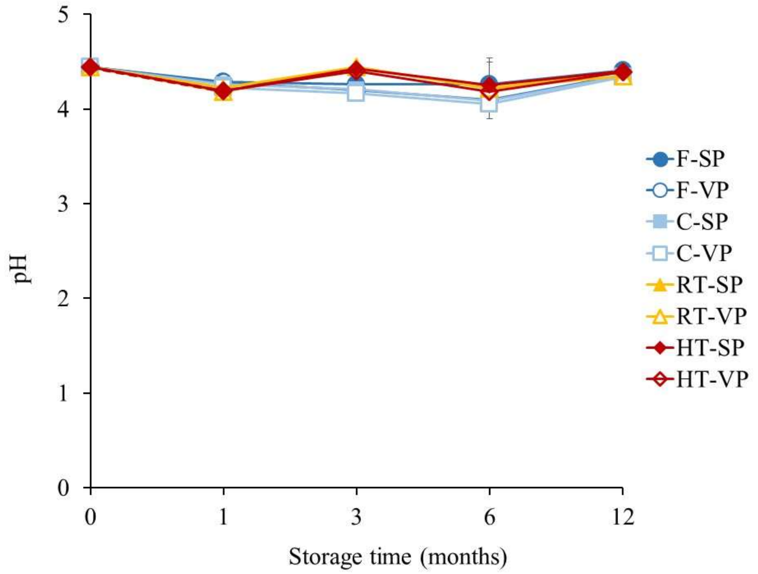


| Sample Code | Temperature | Packaging | Storage time (months) | | | | |
|---|---|---|---|---|---|---|---|
| | | | 0 | 1 | 3 | 6 | 12 |
| **F-SP** | -20 ºC | Standard | 4.44 ± 0.00 | 4.29 ± 0.00 | 4.26 ± 0.01 | 4.26 ± 0.24 | 4.41 ± 0.01 |
| **F-VP** | | Vacuum | 4.44 ± 0.00 | 4.28 ± 0.01 | 4.20 ± 0.02 | 4.10 ± 0.01 | 4.36 ± 0.01 |
| **C-SP** | 4 ºC | Standard | 4.44 ± 0.00 | 4.26 ± 0.01 | 4.21 ± 0.01 | 4.09 ± 0.01 | 4.35 ± 0.02 |
| **C-VP** | | Vacuum | 4.44 ± 0.00 | 4.23 ± 0.00 | 4.17 ± 0.03 | 4.05 ± 0.03 | 4.34 ± 0.00 |
| **RT-SP** | 22 ºC | Standard | 4.44 ± 0.00 | 4.23 ± 0.01 | 4.44 ± 0.03 | 4.22 ± 0.32 | 4.40 ± 0.04 |
| **RT-VP** | | Vacuum | 4.44 ± 0.00 | 4.18 ± 0.01 | 4.44 ± 0.01 | 4.22 ± 0.04 | 4.35 ± 0.06 |
| **HT-SP** | 37 ºC | Standard | 4.44 ± 0.00 | 4.19 ± 0.04 | 4.43 ± 0.03 | 4.25 ± 0.02 | 4.40 ± 0.03 |
| **HT-VP** | | Vacuum | 4.44 ± 0.00 | 4.19 ± 0.01 | 4.40 ± 0.01 | 4.18 ± 0.05 | 4.39 ± 0.02 |

**Figure S7.** pH in FFP samples stored under different storage temperatures and packaging modes. FFP: fermented food product; F-SP: freezing standard packaging; F-VP: freezing vacuum packaging; C-SP: cooling standard packaging; C-VP: cooling vacuum packaging; RT-SP: room temperature standard packaging; RT-VP: room temperature vacuum packaging; HT-SP: high temperature standard packaging; HT-VP: high temperature vacuum packaging. Values are expressed as mean ± SD.

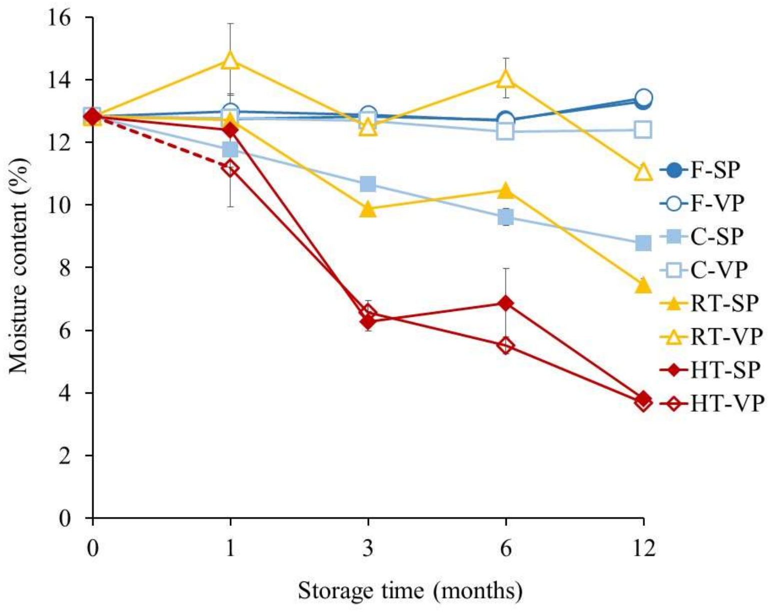


| Sample Code | Temperature | Packaging | Storage time (months) | | | | |
|---|---|---|---|---|---|---|---|
| | | | 0 | 1 | 3 | 6 | 12 |
| **F-SP** | -20 ºC | Standard | 12.84 ± 0.21 | 12.75 ± 0.09 | 12.82 ± 0.13 | 12.73 ± 0.03 | 13.31 ± 0.13 |
| **F-VP** | | Vacuum | 12.84 ± 0.21 | 12.98 ± 0.08 | 12.89 ± 0.12 | 12.69 ± 0.16 | 13.43 ± 0.16 |
| **C-SP** | 4 ºC | Standard | 12.84 ± 0.21 | 11.78 ± 0.24 | 10.66 ± 0.24 | 9.62 ± 0.28 | 8.79 ± 0.06 |
| **C-VP** | | Vacuum | 12.84 ± 0.21 | 12.76 ± 0.07 | 12.68 ± 0.01 | 12.35 ± 0.12 | 12.39 ± 0.22 |
| **RT-SP** | 22 ºC | Standard | 12.84 ± 0.21 | 12.71 ± 0.11 | 9.88 ± 0.03 | 10.48 ± 0.06 | 7.45 ± 0.21 |
| **RT-VP** | | Vacuum | 12.84 ± 0.21 | 14.64 ± 1.15 | 12.49 ± 0.01 | 14.05 ± 0.63 | 11.08 ± 0.21 |
| **HT-SP** | 37 ºC | Standard | 12.84 ± 0.21 | 12.39 ± 1.17 | 6.28 ± 0.31 | 6.86 ± 1.12 | 3.83 ± 0.16 |
| **HT-VP** | | Vacuum | 12.84 ± 0.21 | 11.19 ± 1.25 | 6.57 ± 0.37 | 5.51 ± 0.27 | 3.68 ± 0.06 |

**Figure S8.** Moisture content (%) in FFP samples stored under different storage temperatures and packaging modes. FFP: fermented food product; F-SP: freezing standard packaging; F-VP: freezing vacuum packaging; C-SP: cooling standard packaging; C-VP: cooling vacuum packaging; RT-SP: room temperature standard packaging; RT-VP: room temperature vacuum packaging; HT-SP: high temperature standard packaging; HT-VP: high temperature vacuum packaging. Values are expressed as mean ± SD.
